# Supplementary material for: Identification and functional analysis of non-coding regulatory small RNA FenSr3 in Bacillus amyloliquefaciens LPB-18
Source: PeerJ. 2023 May 15;11:e15236. doi: 10.7717/peerj.15236 (PMC10194069; doi:10.7717/peerj.15236)
Supplement: Supplemental Information 4 [file peerj-11-15236-s004.zip › KO/CK-vs-T1_map/map00945.html]

KEGG PATHWAY: Stilbenoid, diarylheptanoid and gingerol biosynthesis - Reference pathway


|  |  |
| --- | --- |
| **Stilbenoid, diarylheptanoid and gingerol biosynthesis - Reference pathway** |  |

[
Pathway menu
| Organism menu
| Pathway entry
| Show description
| User data mapping
]

Stilbenoids are a group of phenolic compounds, biosynthetically interrelated through their common origin from a C6-C2-C6 intermediate, such as resveratol found in grapes. Stilbenoids can also exist as glycosides (e.g., piceid). Combretastatins are potentially useful stilbenoid natural products with known antitumor activity. Diarylheptanoid is a compound group having phenyl rings at 1,7 positions of n-heptane (C6-C7-C6), such as curcumin found in the ginger family. [6]-Gingerol is a major active component of ginger and has diverse pharmacologic effects.

|  |  |  |
| --- | --- | --- |
| Reference pathway | 184% 150% 122% 100% 82% 67% 55% | 图片下载 |
